# A lack of consideration of a dose–response relationship can lead to erroneous conclusions regarding 100% fruit juice and the risk of cardiometabolic disease

**DOI:** 10.1038/s41430-019-0514-x

**Published:** 2019-10-21

**Authors:** Tauseef A. Khan, Laura Chiavaroli, Andreea Zurbau, John L. Sievenpiper

**Affiliations:** 1 Department of Nutritional Sciences, Faculty of Medicine, University of Toronto, Toronto, ON Canada; 2 Toronto 3D Knowledge Synthesis and Clinical Trials Unit, Clinical Nutrition and Risk Factor Modification Centre, St. Michael’s Hospital, Toronto, ON Canada; 3 Division of Endocrinology and Metabolism, Department of Medicine, St. Michael’s Hospital, Toronto, ON Canada; 4 Li Ka Shing Knowledge Institute, St. Michael’s Hospital, Toronto, ON Canada; 5 Department of Medicine, Faculty of Medicine, University of Toronto, Toronto, ON Canada

**Keywords:** Risk factors, Risk factors, Cardiovascular diseases, Risk factors, Cardiovascular diseases

## Sugar-sweetened beverages and 100% fruit juice: a tale of two dose-response analyses

Excess intake of added sugars especially from sugar-sweetened beverages (SSBs) is associated with increased risk of obesity, type 2 diabetes, cardiovascular disease (CVD) and mortality [1]. Mounting evidence suggests that SSBs contribute a substantial proportion of daily intake of sugars [2] while offering no nutritional benefit; and it is the obesogenic effect of excess calories that increases the risk of cardiometabolic disease and mortality rather than any adverse effect of fructose-containing sugars [3]. The consistent relationship between SSBs and cardiometabolic disease outcomes in several large cohorts has shaped public health guidelines calling for the reduction of SSBs [4] and has resulted in a soda-tax within several countries.

In contrast to SSBs, whole fruits have always been a component of a healthy dietary pattern and its liquid form, 100% fruit juice, is also included as an option to meet the recommended targets for fruit and vegetable intake in some nutrition guidelines globally (https://www.nhs.uk/live-well/eat-well/5-a-day-what-counts/). However, recently there have been attempts to group 100% fruit juices and SSBs as “sugary drinks” [5] as both have similar energy densities: A typical 250 ml of 100% orange juice contains 110 kcal and 26 g of sugar; 250 ml of soda contains 105 kcal and 26.5 g of sugar. While equating SSBs and 100% fruit juice on sugar content makes logical sense, especially when we consider that liquid calories are less likely to be compensated [6], it ignores the fact that 100% fruit juice, in a similar manner to fruit, also provides health-promoting micronutrients such as vitamins, minerals and polyphenols which have health protective effects [7]. The question remains as to whether there is any evidence demonstrating an association between 100% fruit juice and cardiometabolic disease that is similar to that found with SSBs. Seeking a simple binary answer of harm or benefit would be overly simplistic as it disregards the complex relationships that exists between various foods, their nutrient matrix and disease. We propose that the evidence needed to assess if 100% fruit juices are similar to SSBs require us to understand the population level dose–response relationships.

**Fig. 1:**
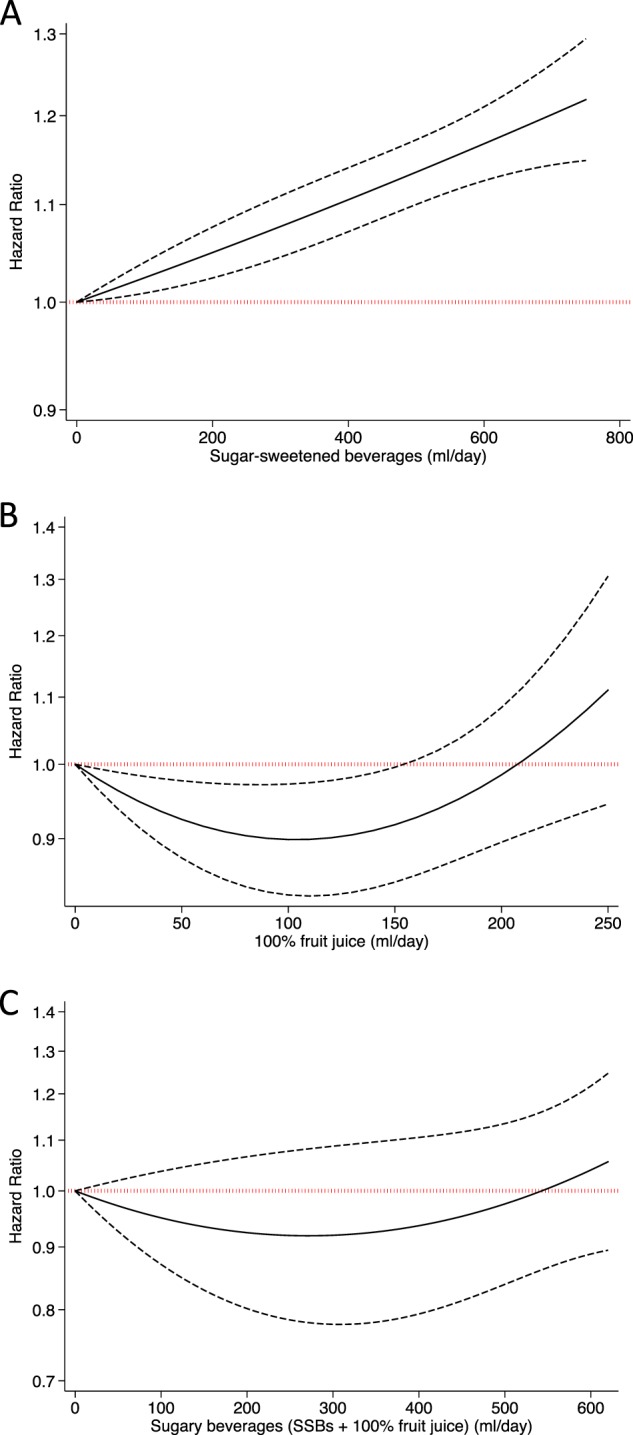
Dose–response relationship of **a**. SSBs with total mortality [9], **b**. 100% fruit juice with CVD incidence [13] and **c**. sugary beverages (SSBs + 100% fruit juice) with total mortality [5]. For each study we modelled the non-linear dose–response using the best fitting second-order fractional polynomial regression using summarised data [8]

Using aggregate data from categorical tables of three separate published studies reporting on disease associations for SSBs, 100% fruit juice or both (“sugary drinks”), we modelled a non-linear dose–response using the best fitting second-order fractional polynomial regression [8]. In Fig. 1a, using data from the Health Professionals’ Follow-up Study and the Nurses’ Health Study, the dose–response association of SSBs with total mortality follows a linear model: [9]. A similar linear dose–response is also found in other prospective cohort studies assessing the relationship between SSBs and CVD mortality [9], type 2 diabetes [10], metabolic syndrome [11] and hypertension [12]. Therefore, studies are correct in reporting disease associations for SSBs as linear analyses or highlight extreme comparisons, which assumes linearity, because the results are consistent with the linear risk curve across the whole dose range.

However, this is not the case with 100% fruit juice. In Fig. 1b, data analysed from a large European cohort [13] demonstrate a non-linear J-shaped curve, revealing a protective association between 100% fruit juice and CVD incidence at moderate doses but indicating harm at higher doses. The curve demonstrates a maximum benefit at doses from 100 to 150 ml/day, which is equal to a small glass of 100% fruit juice. Similar non-linear protective associations at moderate doses for 100% fruit juice consumption are also seen with stroke [13], type 2 diabetes [10], metabolic syndrome [14] and hypertension [15] (there are no studies reporting an association between 100% fruit juice and total mortality). Therefore, reporting linear or extreme comparison analysis that assumes linearity between 100% fruit juice and cardiometabolic disease outcomes would be incorrect. The results from such analyses would only apply at high intakes and the overall conclusions reached would be spurious as they mask any protective associations at moderate doses.

In a recent article by Collins et al. [5], using data from the REGARDS cohort in USA, the authors used a linear analysis and concluded that each additional 12-oz serving of 100% fruit juice per day is associated with higher all-cause mortality. Since 75% of the cohort consumed less than 8-oz daily, the result from this analysis was likely driven by a limited number of extreme consumers of 100% fruit juice. Furthermore, the authors did not provide information on 100% fruit juice consumption to assess a dose–response relationship. Instead, data were reported as categorised energy groups for “sugary beverages”—a combination of 100% fruit juice and SSBs. In Fig. 1c, using data for sugary-beverages, we show that the best-fit relationship, for sugary beverages and total mortality, is nearly J-shaped, suggesting a protective association, albeit non-significant, at moderate doses. Visually, this curve lies in between the linear dose–response of SSBs (Fig. 1a) and non-linear dose–response of 100% fruit juice (Fig. 1b) suggesting an influence of 100% fruit juice at moderate doses. The potential benefit of 100% fruit juice seen at moderate doses may be the result of the range of nutrients and bioactive compounds within the juice. However, the potential for harm at higher doses may be due to the consumption of excess calories outweighing any benefit of the nutrients contained within 100% fruit juice. By combining SSBs and 100% fruit juice, Collins et al. ignored the prevailing independent dose–response curves of each food source. In conclusion, these three examples indicate that SSBs and 100% fruit juice have different dose–response relationships and a lack of consideration of such relationships can lead to inaccurate reporting and erroneous conclusions especially regarding 100% fruit juice and cardiometabolic disease.

## The simple model for sugar-sweetened beverages

When assessing the relationship between food and disease, a linear analysis is routinely carried out as it is the simplest model that can represent the data. For example, if an analysis between a food source of sugars and total mortality shows a significant harmful association in a per-dose analysis, it comes with an implicit assumption that the risk association is linear throughout the dose range and there is no level of exposure at which the association is null or protective. This assumption has important implications for developing guidelines and associated policy for particular foods. As SSBs consistently show an increasing association throughout the investigated dose ranges in large prospective cohort studies, guidelines to reduce its intake is sensible and based upon a demonstrated linear dose–response relationship. However, when the true underlying association is non-linear, as is the case with 100% fruit juice, it is inaccurate to represent the association as a per-dose analysis. Such over-simplistic representation of the overall association that ignores the non-linearity in the dose–disease relationship can easily result in misleading conclusions. The adage attributed to Einstein applies here: “Everything must be made as simple as possible, but not one bit simpler” [16]. In the case of a non-linear association, the “dose–response” model itself is the *simple model* of the underlying association; forcing an even *simpler* linear model that the data do not support would be a fallacy.

## Dose–response relationship in nutrition research: the next frontier

Non-linear association are well established for essential nutrients following a U-shaped relationship curve in which low dose is harmful due to deficiency, intermediate dose (or replete states) elicit positive health effects and high doses are harmful because of toxicity (https://ods.od.nih.gov/Health_Information/Dietary_Reference_Intakes.aspx). Foods, which contain essential nutrients, can thus be reasonably expected to follow non-linear associations with health outcomes as well. Having said that, the reporting of non-linear dose–response association the literature for major food sources investigating cardiometabolic disease leaves much to be desired. A majority of prospective cohort studies investigating diet and disease start with linearity as the primary model and denote harm or protective associations to various foods without investigating the subtle relationships of disease with dose ranges—which might be non-linear. Their conclusions are focused upon a linear dose–response or an extreme comparison of categorical groups (e.g. quantiles) that also implies linearity, while often the associated categorical tables suggest non-linearity. Although the linear association might hold true for extreme intakes, ignoring the nuanced relationship of the dose range is one reason why the science of nutrition is facing many challenges especially in reporting numerous contradictory results using linear analyses [17]. These results might simply be a result of applying the incorrect linear model to a non-linear association, an inability to assess non-linearity due to the lack of power, or a difference in population dose ranges. In the latter case, depending upon the dose range observed, one can expect divergent results from studies conducted in different populations as each would be assessing a limited portion of a true non-linear curve.

A complete dose–response analysis in a prospective cohort study should include exploring doses and dose ranges that are protective or harmful, with threshold or plateau effects, and examining differences between moderate and high intakes. In fact, further analyses can be performed to see if dose ranges differ for populations, ethnicities, ages, health status, sex, demographics or any other important characteristics. The dose–response analysis can also drive insights into mechanisms by exploring why such non-linear associations exist in the first place: what drives the neutral, protective and harmful effects; why are there different effects at low, moderate and high doses; does excessive calories overwhelm the protective effect of beneficial nutrients; how does the nutrient matrix within foods affect dose–response relationships; and is there an underlying biological or evolutionary mechanism for such non-linear effects, e.g. hormesis [18]. The importance of dose-response analyses to further the field of nutrition cannot be understated. In fact, writing in the journal *Science* the Global Panel on Agriculture and Food Systems for Nutrition in their global research agenda for nutrition highlighted the need for a “better understanding of dose–response relationships” of foods which will enable us to agree on what constitutes a healthy diet and denoted it as one of the top ten research priorities for the upcoming decade [19].

As the case of 100% fruit juice above exemplifies, it is vitally important to explore and report the full dose–response in prospective cohort studies rather than a linear analysis. In-fact many nutrients and foods have reported non-linear relationships with cardiometabolic disease outcomes including fruit [20], nuts [20], salt [21], coffee [22], dairy [20], unprocessed red meat [20], whole grains [20] and total carbohydrates [23]. However, only a few authors have attempted to make informed interpretations of non-linear dietary data in a population context for cardiometabolic disease [21, 23].

There are now several methods to study underlying non-linear dose–response associations in prospective cohort studies using restricted cubic splines, fractional polynomials and categorical modelling [24]. New methods are also available to combine dose–response analyses in meta-analysis [25]. While it’s true that modelling dose–response is complex, and reporting standards do not yet give any concrete instructions on how best to report non-linear dose–response associations, we simply cannot ignore the elephant in the room i.e. the realisation that the relationship might not be linear.

## Conclusion

The concern regarding dietary sugars continues to grow and 100% fruit juice is now being discouraged by health agencies due to its high sugar content [4]. Even public perception of 100% fruit juice has been shifting to equate it with SSBs (https://www.nytimes.com/2018/07/07/opinion/sunday/juice-is-not-healthy-sugar.html). We contend that this issue has arisen due to the lack of consideration for the dose–response association between 100% fruit juice and cardiometabolic disease. Investigators of prospective cohort studies studying beverages, but also other important food sources, should consider modelling dose–response associations with disease to see if they are non-linear. If so, then the identification of specific dose ranges or cut-offs for benefit and harm would have important implications for dietary guidelines and public policy. Failing to do so will only perpetuate the misinterpretation of the results in nutrition research leading to inaccurate conclusions regarding relationships between foods and health.
